# Symptomatic Anterior Cervical Osteophyte Causing Dysphagia: Case Report, Imaging, and Review of the Literature

**DOI:** 10.7759/cureus.473

**Published:** 2016-02-02

**Authors:** Yi-Ren Chen, Kwang Sung, Suzanne Tharin

**Affiliations:** 1 Department of Neurosurgery, Stanford University School of Medicine; 2 Department of Otolaryngology – Head & Neck Surgery, Stanford University School of Medicine

**Keywords:** cervical osteophyte, osteophytectomy

## Abstract

Anterior cervical osteophytes are found in 20-30% of elderly patients. Rarely, severe osteophytes can cause dysphagia, dysphonia, and dyspnea. Here, we illustrate a case of severe dysphagia caused by a large post-traumatic osteophyte with oropharyngeal swallow study showing a significant mass effect on the pharynx and resolution following osteophytectomy. We also review the literature regarding the etiology, diagnosis, and treatment of symptomatic anterior cervical osteophytes.

## Introduction

Greater than 75% of people aged 65 and older have varying degrees of cervical spine degenerative changes, including hypertrophic anterior cervical osteophytes [1-3]. Specifically, anterior cervical osteophytes have a prevalence of 20-30% in the elderly population [4]. Causes of cervical osteophytes include diffuse idiopathic skeletal hyperostosis, ankylosing spondylitis, degenerative changes, and prior trauma, including surgery [5]. Anterior cervical osteophytes are generally asymptomatic; however, in rare cases, they can lead to dysphagia, dysphonia, and dyspnea [6-7]. Such symptoms are generally correlated with the size of the hypertrophic spurs [8]. Here, we present a case of a 63-year-old man presenting with a large anterior osteophyte causing severe dysphagia, whose symptoms resolved following cervical osteophytectomy. 

## Case presentation

A 63-year-old man with a history of neck trauma resulting in vocal cord injury ten years prior presented with two years of progressive dysphagia. He was unable to swallow solid foods, and could tolerate only a puree and liquid diet. He had no dysphonia or dyspnea but did have some odynophagia on swallowing. He was otherwise healthy with no other medical history. Informed patient consent was obtained for treatment.

**Figure 1:**
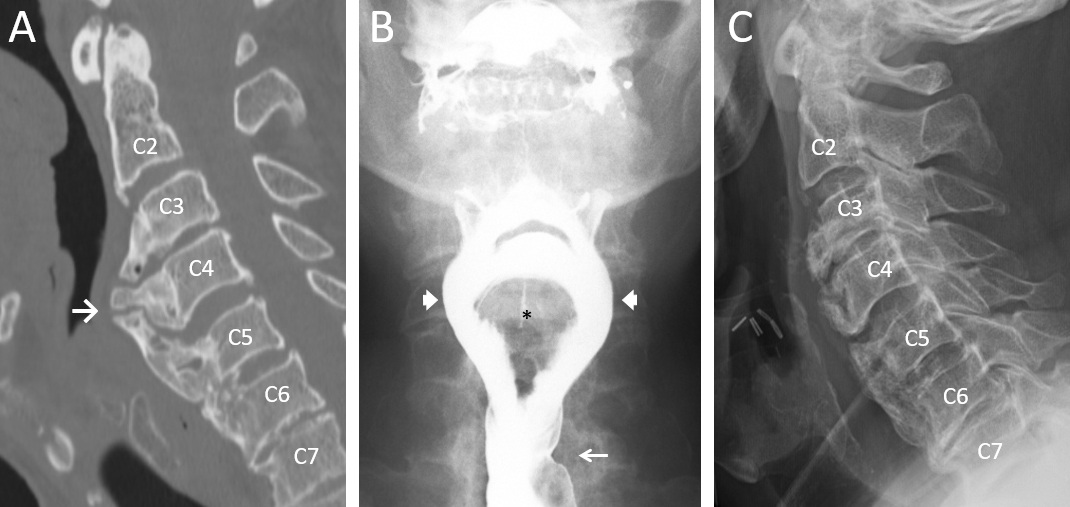
Anterior cervical osteophyte before and after osteophytectomy

Computed tomography (CT) of the cervical spine showed a large anterior osteophyte spanning from C3 to C6 and measuring 19 mm at its greatest anteroposterior extent (Figure 1A, arrow). An oropharyngeal swallow study showed a significant mass effect on the pharynx and splitting of the contrast into two tracts (arrowheads) around the osteophyte (asterisk) during active swallow on the anteroposterior view (Figure 1B). In addition, cricopharyngeus (CP) hypertrophy with mild narrowing of the upper esophageal sphincter was observed (thin arrow). Aspiration of liquids and, to a lesser extent, pureed foods, was noted on swallow studies.

The patient underwent an anterior cervical osteophytectomy with concomitant CP myotomy. Bone wax was used intra-operatively to prevent bone regrowth [9]. A postoperative lateral plain film showed the removal of the anterior osteophyte (Figure 1C). The patient reported significant improvement of dysphagia immediately postoperatively and complete resolution of symptoms at his one-month follow-up. At his one-year follow-up, the patient continued to report resolution of his symptoms with normal swallow function. Of note, NSAIDs were administered postoperatively to prevent osteophyte regrowth [10].

## Discussion

Osteophytes can form at any cervical level but are most common at C5-6 and C6-7, likely due to greater load-bearing and mobility [11]. Dysphagia in patients with anterior osteophytes may be explained by the fact that the esophagus rests on the anterior border of the cervical vertebrae from C4-7. Etiologies of dysphagia include a large osteophyte causing significant mechanical compression, a smaller osteophyte causing obstruction at the level of the cricoid cartilage, and inflammation secondary to local mass effect [12-15].

Most anterior cervical osteophytes are asymptomatic; however, they are not an uncommon cause of dysphagia. Interestingly, one study in the veteran population showed anterior cervical bony protrusion in 10.6% of patients over 60 years of age undergoing dysphagia evaluation, suggesting that the incidence of cervical osteophytes causing dysphagia is higher than recognized in the medical community, and underscoring their place in the differential diagnosis of dysphagia [16].

The diagnosis of cervical osteophytes may be accomplished with a CT scan, which can clearly define the bony anatomy. A barium swallow is essential to confirm the presence of esophageal compression by the cervical osteophyte. Magnetic resonance imaging (MRI) may also be considered in patients with additional symptoms, such as dyspnea and dysphonia, to identify any soft tissue damage, perforations, or superinfection. MRI is also useful to evaluate any concomitant central or foraminal cervical stenosis that the surgeon may wish to address at the time of osteophytectomy.

The mainstay of treatment for cervical osteophytes is conservative, including anti-inflammatory medications and diet modification [17]. However, for patients in whom conservative management has failed, operative intervention may be considered. Some authors argue that cervical osteophytectomy should be considered in all patients with cervical osteophytes causing chronic dysphagia and dyspnea because of possible progression to acute respiratory distress. Maiuri, et al. reported a case of a patient with a two-year history of dysphagia who suffered sudden severe respiratory distress requiring emergency tracheotomy [18].

Surgery for cervical osteophytes has good long-term outcomes. Our patient continued to have symptomatic relief at his one-year follow-up. This is in keeping with a prior series published by Urrutia, et al. showing no recurrence of dysphagia and minimal radiographic regrowth at one to nine year (average 59.8 months) follow-up evaluations [19]. A second series published by Miyamoto, et al. reported immediate postoperative relief of dysphagia in all patients, but noted some radiographic recurrence at six‑ to 13-year follow-up evaluations (mean: nine years), with an average rate of regrowth of 1 mm/year [20]. Five out of seven of their patients remained asymptomatic, but two had moderate dysphagia 10 and 11 years after surgery, suggesting a low rate of late symptomatic recurrence [20].

## Conclusions

Large anterior cervical osteophytes are a potential cause of dysphagia. The key imaging study is a barium swallow. Patients with significant symptoms who fail conservative management should be considered for osteophytectomy. Outcomes following osteophytectomy are very favorable. 
